# Author Correction: The mycobacterial glycoside hydrolase LamH enables capsular arabinomannan release and stimulates growth

**DOI:** 10.1038/s41467-024-51724-9

**Published:** 2024-08-21

**Authors:** Aaron Franklin, Vivian C. Salgueiro, Abigail J. Layton, Rudi Sullivan, Todd Mize, Lucía Vázquez-Iniesta, Samuel T. Benedict, Sudagar S. Gurcha, Itxaso Anso, Gurdyal S. Besra, Manuel Banzhaf, Andrew L. Lovering, Spencer J. Williams, Marcelo E. Guerin, Nichollas E. Scott, Rafael Prados-Rosales, Elisabeth C. Lowe, Patrick J. Moynihan

**Affiliations:** 1 School of Biosciences, University of Birmingham, Birmingham, UK; 2 Department of Preventive Medicine, Public Health and Microbiology, School of Medicine, Universidad Autonoma de Madrid, Madrid, Spain; 3 Structural Glycobiology Laboratory, Department of Structural and Molecular Biology, Molecular Biology Institute of Barcelona, Spanish National Research Council, Barcelona Science Park, c/Baldiri Reixac 10-12, Tower R, 08028 Barcelona, Catalonia Spain; 4 School of Chemistry and Bio21 Molecular Science and Biotechnology Institute, University of Melbourne, Parkville, VIC Australia; 5 Structural Glycobiology Laboratory, Department of Structural and Molecular Biology; Molecular Biology Institute of Barcelona (IBMB), Spanish National Research Council (CSIC), Barcelona, Catalonia Spain; 6 Department of Microbiology and Immunology, University of Melbourne at the Peter Doherty Institute for Infection and Immunity, Melbourne, VIC Australia; 7 Newcastle University Biosciences Institute, Medical School, Newcastle University, Newcastle upon Tyne, UK

**Keywords:** Pathogens, Polysaccharides, Tuberculosis

Correction to: *Nature Communications* 10.1038/s41467-024-50051-3, published online 09 July 2024

The original version of this Article contained an error in Fig. 3, in which the *X*-axis was incorrectly labelled in panel d. This has been corrected in both the PDF and HTML versions of the Article.

