# Supplementary material for: The mycobacterial glycoside hydrolase LamH enables capsular arabinomannan release and stimulates growth
Source: Nat Commun. 2024 Jul 9;15:5740. doi: 10.1038/s41467-024-50051-3 (PMC11233589; doi:10.1038/s41467-024-50051-3)
Supplement: Supplementary file 1 — Supplementary Information [file 41467_2024_50051_MOESM1_ESM.pdf]

Supplementary Materials for:

The mycobacterial glycoside hydrolase LamH enables capsular arabinomannan release and stimulates growth

Aaron Franklin[1], Vivian C. Salgueiro[2], Abigail J. Layton[1], Rudi Sullivan[1], Todd Mize[1], Lucía Vazquez[2], Samuel T. Benedict[1], Sudagar S. Gurcha[1], Itxaso Anso[3], Gurdyal S. Besra[1], Manuel Banzhaf[1], Andrew L. Lovering[1], Spencer J. Williams[4], Marcelo E. Guerin[5], Nichollas E. Scott[6], Rafael Prados-Rosales[2], Elisabeth C. Lowe[7]*, Patrick J. Moynihan[1]*

1. School of Biosciences, University of Birmingham, Birmingham, U.K., B15 2TT
2. Department of Preventive Medicine, Public Health and Microbiology. School of Medicine. Universidad Autonoma de Madrid, 28029 Madrid, Spain
3. Structural Glycobiology Laboratory, Biocruces Health Research Institute, Barakaldo, Bizkaia, 48903, Spain.
4. School of Chemistry and Bio21 Molecular Science and Biotechnology Institute, University of Melbourne, Parkville, Victoria 3010, Australia
5. Structural Glycobiology Laboratory, Department of Structural and Molecular Biology; Molecular Biology Institute of Barcelona (IBMB), Spanish National Research Council (CSIC), Barcelona Science Park, c/Baldiri Reixac 4-8, Tower R, 08028 Barcelona, Catalonia, Spain.
6. Department of Microbiology and Immunology, University of Melbourne at the Peter Doherty Institute for Infection and Immunity, Melbourne 3000, Australia
7. Newcastle University Biosciences Institute, Medical School, Newcastle University, Newcastle upon Tyne, U.K., NE2 4HH

*To whom correspondence should be addressed:

Patrick Moynihan, p.j.moynihan@bham.ac.uk

Elisabeth Lowe, elisabeth.lowe@ncl.ac.uk

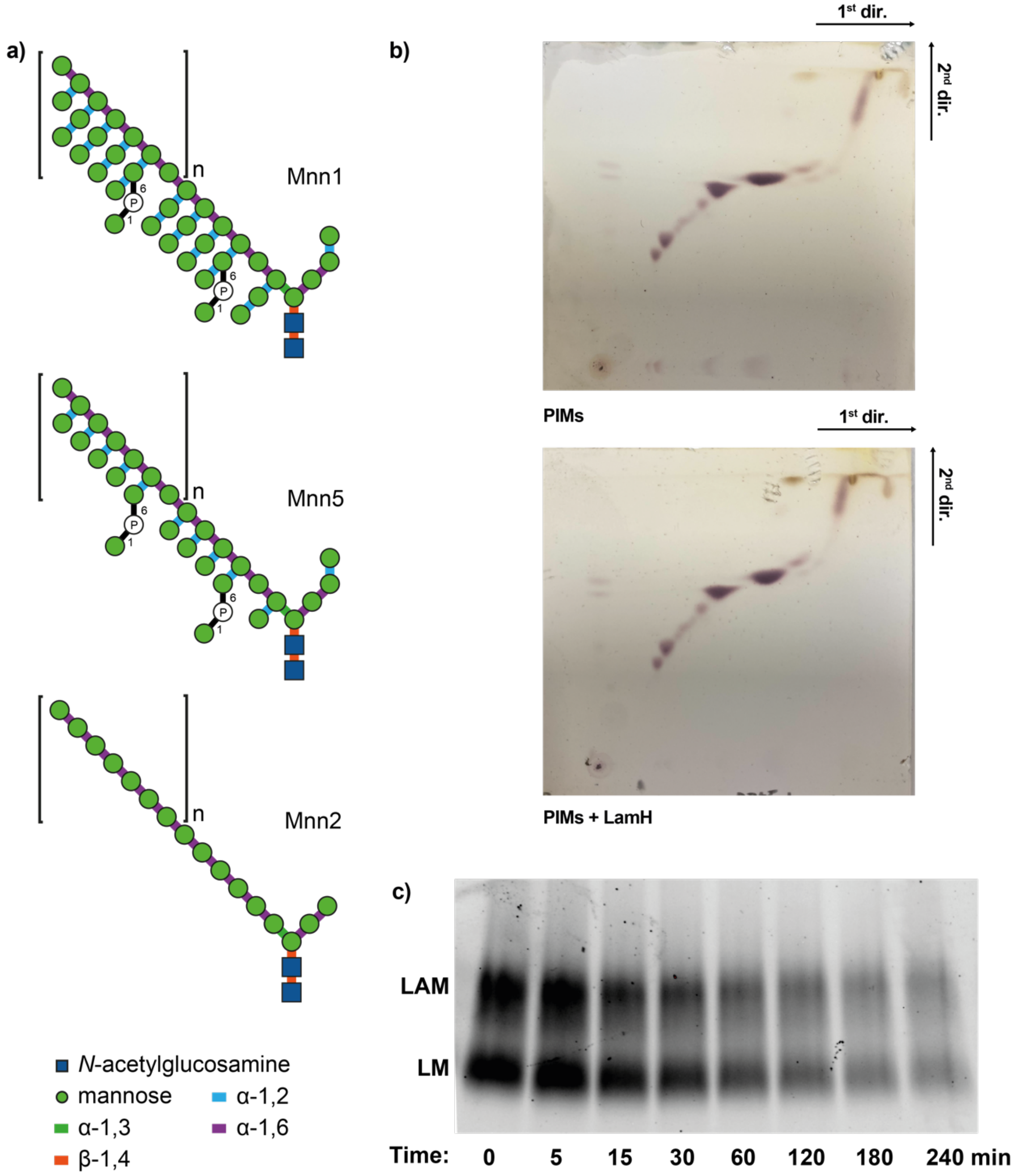

Supplementary Figure 1. LamH substrate specificity. A) Structure of the mannans purified from mutant strains of *S. cerevisiae*. **B)** Representative two-dimensional TLC analysis of polar lipids from *M. bovis* BCG Danish wild-type with and without the addition of LamH. Equal volumes were loaded onto the TLC and separated in the first direction in solvent system E1: chloroform:methanol:water (60:30:6 v/v/v) and in the second direction in solvent system E2: chloroform:acetic acid:methanol: water (40:25:3:6 v/v/v/v). TLCs were visualised by staining with oricinol and charring. **C)** Example SDS-PAGE time-course data for Fig. 2d. Purified LAM/LM was incubated with LamH at 37 °C, and aliquots were taken at the given time points. The reaction mixture was heat-inactivated at 100 °C for 10 min. Subsequently, the time points were separated by SDS-PAGE and stained with Pro-Q Emerald. The gel was visualised by fluorescence imaging at 300 nm on a Bio-Rad Gel Doc XR+. Bands were quantitated using the Bio-Rad ImageLab v6.1 software package, and these data are presented in Fig. 2d. The experiment was repeated with three biological replicates. Source data are provided in the source data file.

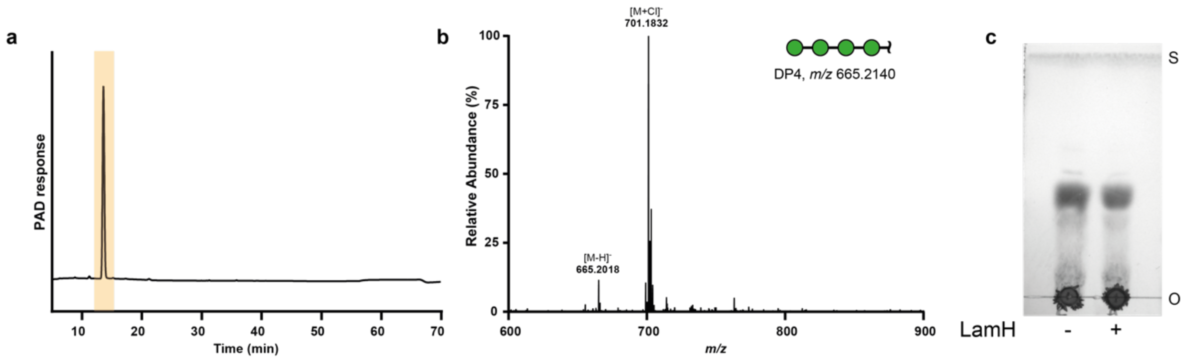

Supplementary Figure 2. Rv0365c substrate specificity. Mannotetraose (50 µM) purified from an enzymatic digest of mnn2 was analysed by IC-MS. **a)** PAD response with mannotetraose peak highlighted. **b)** An extracted ion chromatogram of the highlighted peak shows the detection of mannotetraose ions. **c)** LamH was incubated with capsular glucan derived from the Δ*lamH* strain for 18 h. The reactions were separated by TLC (2:1:1, butanol: acetic acid: water) and visualised by staining with orcinol. Representative data for three biological replicates are presented. Source data are provided in the source data file.

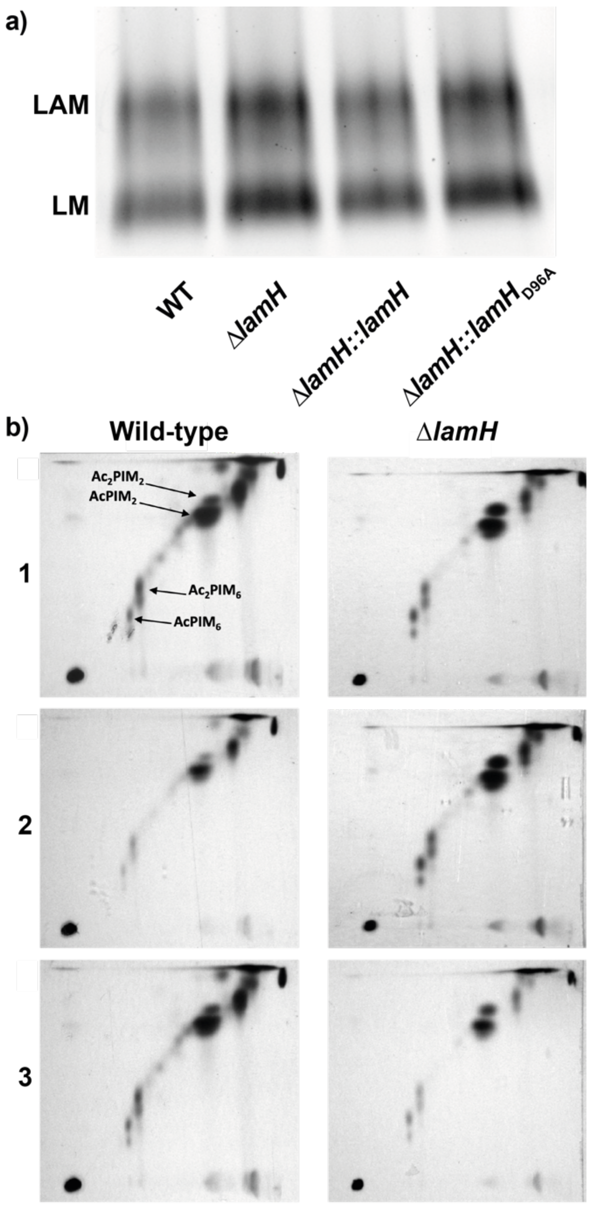

Supplementary Figure 3. Lipoglycan analysis. a) Representative LM/LAM SDS-PAGE gel from 3 biological replicates of selected *M. bovis BCG* Danish strains. The gel was imaged as described sabove. **b)** Two-dimensional TLC analysis of ^{14}C labelled polar lipids from *M. bovis* BCG Danish wild-type and Δ*lamH* mutants. Equal counts of extract were loaded onto the TLC and separated in the first direction in solvent system E1: chloroform:methanol:water (60:30:6 v/v/v) and in the second direction in solvent system E2: chloroform:acetic acid:methanol: water (40:25:3:6 v/v/v/v). TLCs were visualised by exposure to X-ray film by autoradiography. PIMs are annotated as per Driessen *et al.* (2009). Three biological replicates are presented. Source data are provided in the source data file.

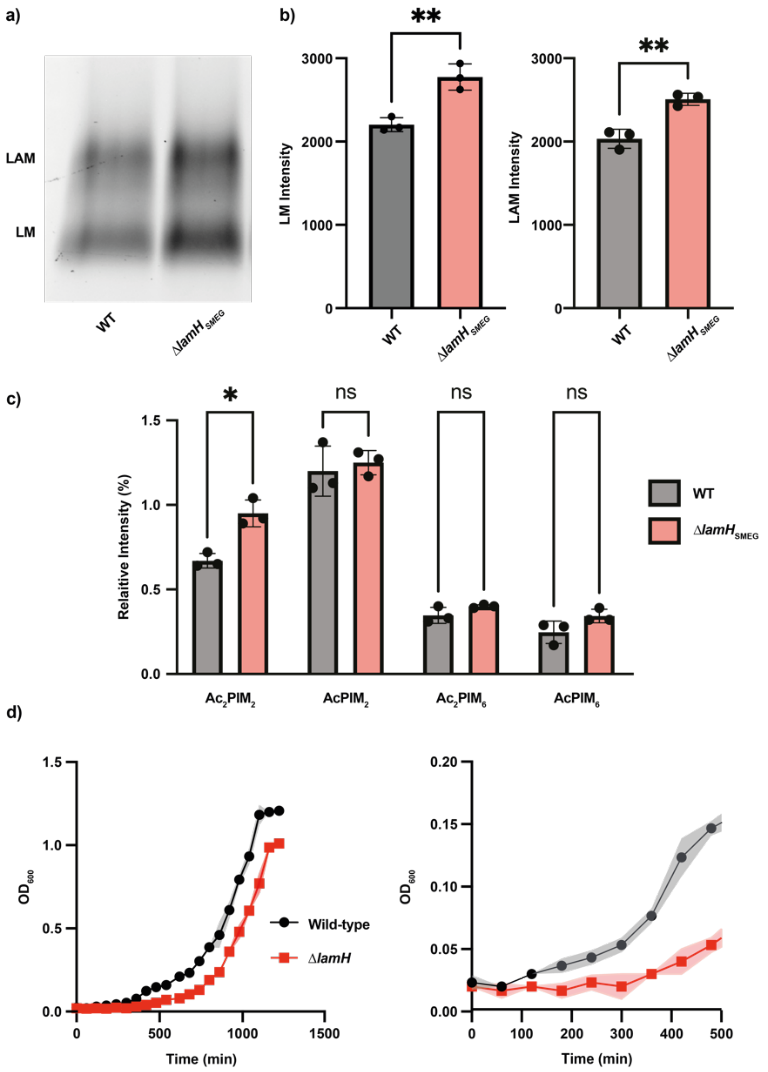

Supplementary Figure 4. LamH function is conserved in *M. smegmatis*. a) LM/LAM was purified from an equal number of cells and was analysed by SDS-PAGE and Pro-Q Emerald staining with three biological replicates. The gel was visualised by fluorescence at 300 nm on a Bio-Rad Gel Doc XR+. **b)** Fluorescence was determined for three biological replicates for both LM and LAM using Bio-Rad ImageLab v6.1 software package and is presented with error bars indicating standard deviation. Significance was determined by an unpaired t-test. **$P < 0.01$. **c)** The indicated *M. smegmatis* strains were grown in 7H9 media in the presence of (1-^{14}C) acetic acid, sodium salt until OD_{600} = 0.6. Polar lipids were analysed via two-dimensional TLC and visualised by exposure to X-ray film by autoradiography. The relative intensity of each PIM species is reported. Significance determined by one-way ANOVA. *$P < 0.05$. **d)** Growth kinetics of the indicated *M. smegmatis* strains are shown on the left. n = 3 biological replicates. The shaded area represents 95% confidence intervals of the three replicates. OD_{600} was measured every 60 minutes until the stationary phase was reached. On the right, the time points 0 to 500 minutes are shown. At this point, the transition into logarithmic growth occurs. Source data are provided in the source data file.

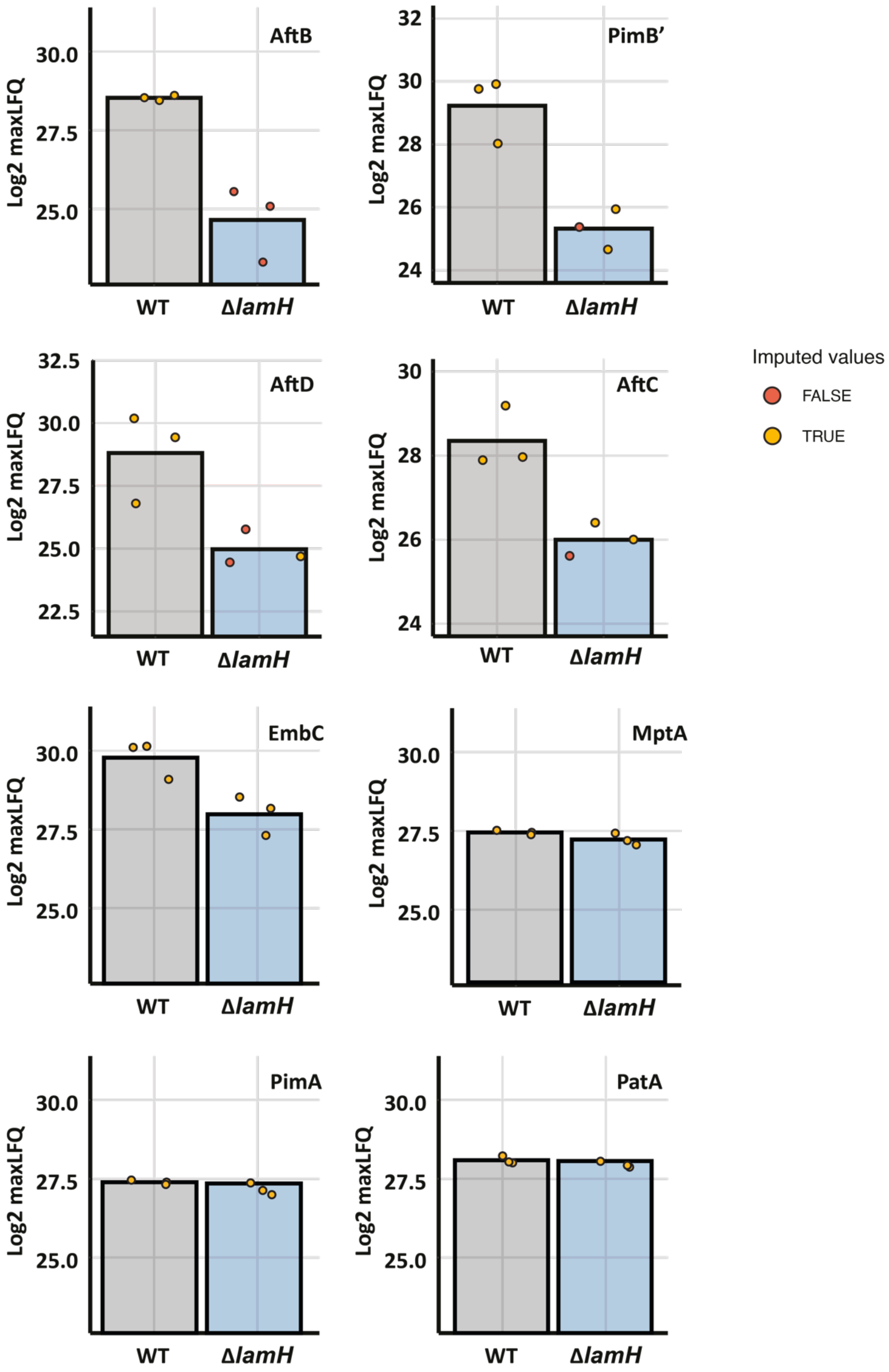

Supplementary Figure 5. Quantitation of Δ*lamH* proteomics. Quantitation of individual protein levels observed across biological replicates supports the reduction in the abundance of proteins associated with the LAM biosynthetic pathway within Δ*lamH*. Imputed values are denoted in red, while experimentally observed values are denoted in orange. Source data are provided in the source data file.

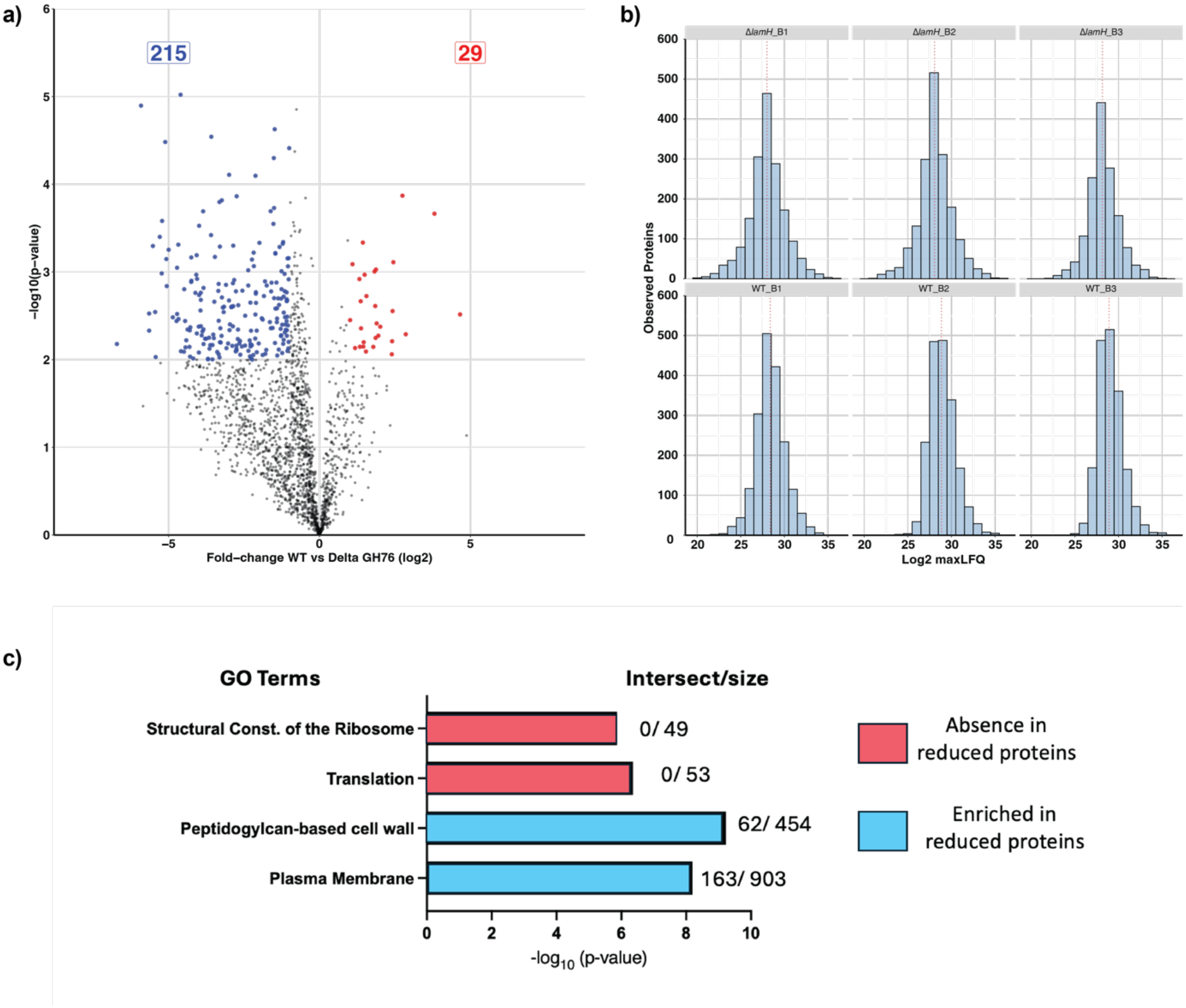

Supplementary Figure 6. Δ*lamH* proteomic analysis. a) Based on a threshold of P < 0.01 and >±1-fold change, we observe 215 proteins decreasing in abundance and 29 increased within Δ*lamH* out of the total 1916 proteins identified. **b)** For each biological replicate, the observed LFQ (Log_2) distributions are provided, demonstrating that observed medians spread in the observed LFQ values (observed maximum and minimum values) as well as the total numbers of identified protein groups across each biological replicate are similar. The median of each distribution is shown in red. c) Gene Ontology (GO)-based enrichment of proteins involved in various biological processes analysed using Fisher's exact enrichment analysis. Percentages represent the proportions of proteins enriched in each category compared to the observable proteome. Underlying data are presented in Supplementary Table 1. Source data are provided in the source data file.

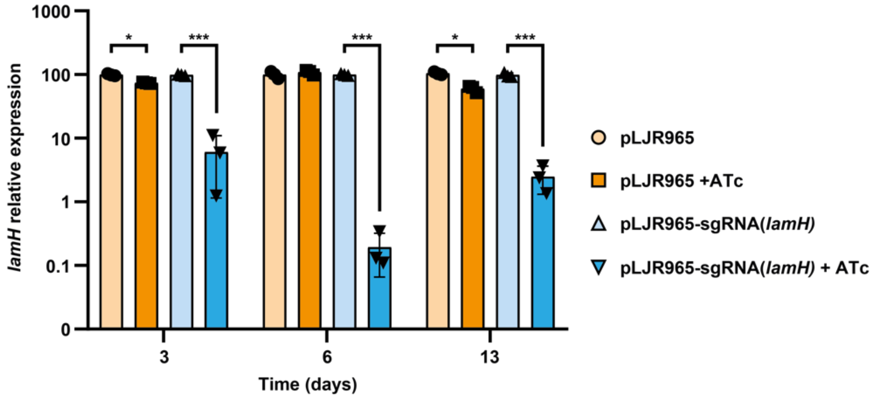

Supplementary Figure 7. CRISPRi-induced knock-down of *lamH* expression in *M. tuberculosis* H37Rv. Quantification (mean ± S.E.M., n = 3 biological replicates) of *lamH* mRNA levels by RT-qPCR. Strains were grown ± ATc for ~3 generations before collecting RNA at the indicated time points. Statistical significance analysis was carried out by running multiple t-tests. Discovery was determined using the Two-stage linear step-up procedure of Benjamini, Krieger and Yekutieli, with Q = 1%. Each row was analysed individually without assuming a consistent SD. Number of t-tests: 3. *P < 0.05; ***P < 0.0001. Source data are provided in the source data file.

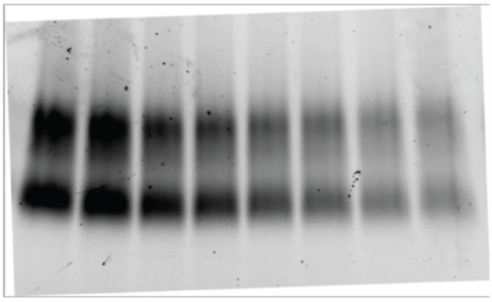

Figure 2f (replicate 1)/Extended data Figure 1c

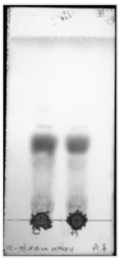

Supp. Figure 2

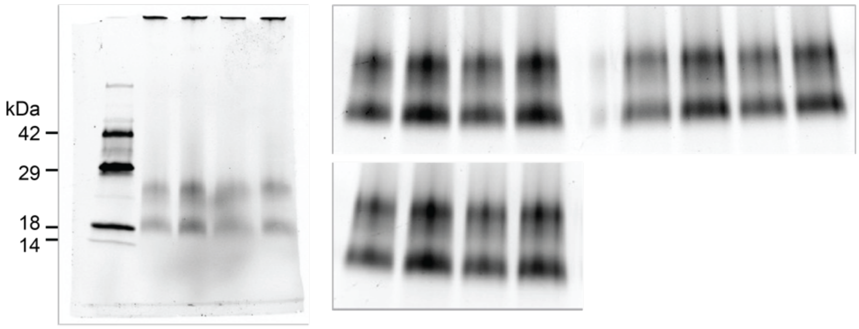

Figure 3a and b/Supplementary Figure 2a

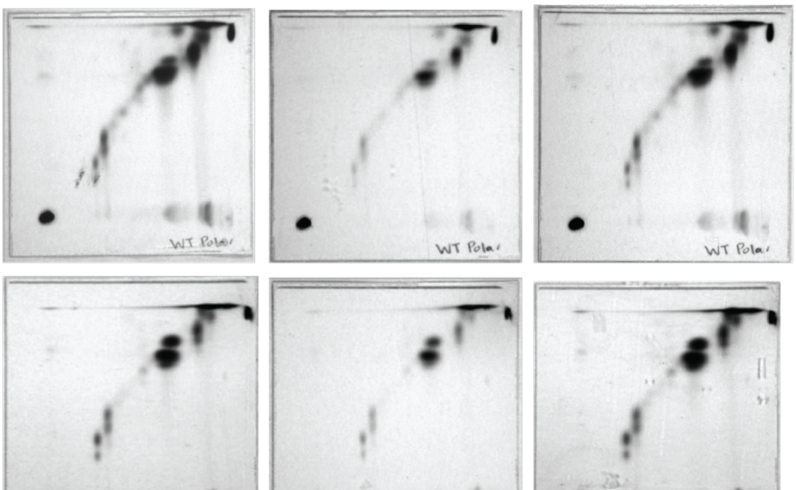

Figure 3e replicates/Extended data Fig 2b

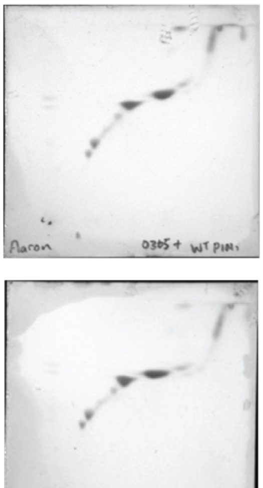

Supplementary Fig. 1b

Supplementary Figure 8. Unprocessed TLCs and SDS PAGE gels.

Supplementary Table 1. Peak identification for Figure 2h. The peak label, identified structure, chemical formula, calculated mass, and observed *m/z* are presented.

Peak Label	Structure	Formula	Calc. *m/z*	*m/z* Obs.	Δ
**A**	phosphoglycerol - 2 x H_2O	$C_3H_4O_4P$	134.98	134.98	0
**B**	phosphoglycerol - H_2O	$C_3H_6O_5P$	152.99	152.99	0
**C**	palmytic acid	$C_{16}H_{31}O_2$	255.23	255.23	0
**D**	tuberculostearic acid	$C_{19}H_{37}O_2$	297.28	297.28	0
**E**	PIM - H_2O	$C_{12}H_{20}O_{13}P$	403.06	403.06	0
**F**	phophogylcerol + tuberculostearic acid - H_2O	$C_{22}H_{42}O_6P$	433.27	433.27	0
**G**	PIM_2 - H_2O	$C_{34}H_{60}O_{19}P$	803.35	803.35	0
**H**	PIM_2	$C_{34}H_{62}O_{20}P$	821.35	821.35	0
**I**	PIM_2 - 2 x H_2O	$C_{37}H_{64}O_{20}P$	859.37	859.37	0
**J**	PIM_2 - H_2O	$C_{37}H_{66}O_{21}P$	877.39	877.39	0
**K**	AcPIM - palmytic acid	$C_{50}H_{92}O_{17}P$	995.61	995.61	0
**L**	$AcPIM_2$ - tuberculostearic acid	$C_{53}H_{96}O_{22}P$	1115.61	1115.61	0
**M**	$AcPIM_2$ - palmytic acid - H_2O	$C_{56}H_{102}O_{22}P$	1157.66	1157.66	0
**N**	$AcPIM_2$ - palmytic acid	$C_{56}H_{104}O_{23}P$	1175.67	1175.67	0
**O**	AcPIM	$C_{66}H_{124}O_{19}P$	1251.85	1251.84	-0.01
**Parental :**	$AcPIM_2$	$C_{72}H_{134}O_{24}P$	1413.90	1413.90	0

Supplementary Data

Supplementary Data 1. DDA protein level LFQ analysis of *M. bovis* BCG Danish Δ*lamH* compared to WT. a) Non-imputed data **b)** Imputed data The Perseus processed MSfragger search results for the protein analysis of three biological replicates of strains WT and Δ*lamH* are provided. For each identified protein, the log2 LFQ protein values, the t-test information including the -log10(p-value), the difference in the mean between the groups and if the resulting p-values are below the multiple hypothesis corrected *p*-value is provided. The protein score, number of peptides identified, and protein length are provided for each protein. **c)** GO-based enrichment of proteins involved in various biological processes analysed using Fisher's exact enrichment analysis.

Supplementary Data 2. List of genome accessions used for building a custom Mycobacteriales BLAST database.
