# Supplementary material for: The mycobacterial glycoside hydrolase LamH enables capsular arabinomannan release and stimulates growth
Source: Nat Commun. 2024 Jul 9;15:5740. doi: 10.1038/s41467-024-50051-3 (PMC11233589; doi:10.1038/s41467-024-50051-3)
Supplement: Supplementary file 3 — Description of Additional Supplementary Files [file 41467_2024_50051_MOESM3_ESM.pdf]

Description of Additional Supplementary Files

Supplementary Data 1. DDA protein level LFQ analysis of *M. bovis* BCG Danish Δ*lamH* compared to WT. a) Non-imputed data **b)** Imputed data The Perseus processed MSfragger search results for the protein analysis of three biological replicates of strains WT and Δ*lamH* are provided. For each identified protein, the log2 LFQ protein values, the t-test information including the -log10(p-value), the difference in the mean between the groups and if the resulting p-values are below the multiple hypothesis corrected *p*-value is provided. The protein score, number of peptides identified, and protein length are provided for each protein. **c)** GO-based enrichment of proteins involved in various biological processes analysed using Fisher's exact enrichment analysis.

Supplementary Data 2. List of genome accessions used for building a custom Mycobacteriales BLAST database.
