# Supplementary figures and images for: The mycobacterial glycoside hydrolase LamH enables capsular arabinomannan release and stimulates growth
Source: Nat Commun. 2024 Jul 9;15:5740. doi: 10.1038/s41467-024-50051-3 (PMC11233589; doi:10.1038/s41467-024-50051-3)

**
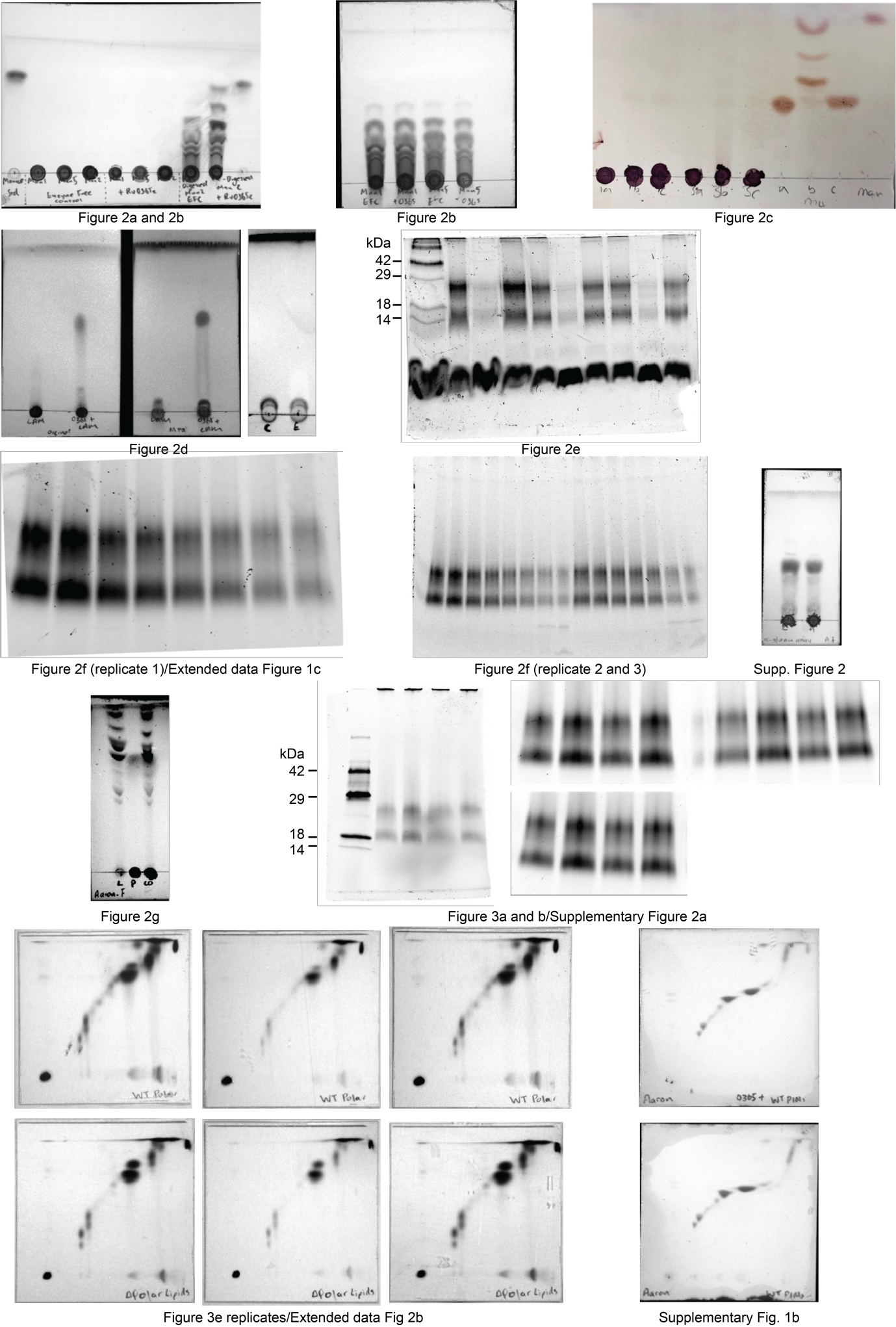
**

**Unprocessed TLCs and SDS PAGE gels.**

Supplement: Supplementary file 7 — Source data [file 41467_2024_50051_MOESM7_ESM.zip › Source Data File_BlotsGels.docx]
